# Assessment of Knowledge and Attitudes Toward Children With Autism Spectrum Disorder Among Undergraduate Nursing Students

**DOI:** 10.7759/cureus.55829

**Published:** 2024-03-08

**Authors:** Hawa M Alabdulaziz, Salmah A Alghamdi, Asala M Aladwani, Rawan A Alharthi, Layla K Almadani, Khadijah A Niyazi, Yara M Alharbi

**Affiliations:** 1 Maternity and Children Department/Pediatric Nursing, King Abdulaziz University, Jeddah, SAU; 2 Maternity and Child Health Department, King Abdulaziz University, Jeddah, SAU; 3 Radiology Department, King Fahad Armed Forces Hospital, Jeddah, SAU; 4 Emergency Department, King Abdullah Medical Complex Jeddah, Jeddah, SAU; 5 Pediatric Ward, King Abdullah Medical Complex Jeddah, Jeddah, SAU; 6 Pediatric Emergency Department, Maternity and Children Specialist Hospital, Jeddah, SAU; 7 Adult Oncology, Hematology, and Bone Marrow Transplantation Department, King Faisal Specialist Hospital & Research Centre, Jeddah, SAU

**Keywords:** asd, attitudes, knowledge, nursing students, autism

## Abstract

Background

There has been a dramatic increase in the incidence and prevalence rate of autism globally. This increase could be correlated to the increase in the awareness level and understanding of the disorder among healthcare providers and autistic children's families.

Aim

This study aimed to assess undergraduate nursing students' knowledge about and attitudes toward children with autism spectrum disorder (ASD).

Methods

A cross-sectional quantitative study design was used. A convenience sample of 235 undergraduate nursing students from all levels was obtained. Data were collected using a questionnaire. Descriptive analysis (including mean, median, and standard deviation) was conducted. Inferential analysis was conducted to identify the relation between participants' demographic data and the subscales.

Results

The majority of the studied sample was aged 20 years or over (n=194, 82.55%) and was from the second year of undergraduate nursing school (n=120, 51.1%). The total mean score of the overall knowledge levels among nursing students at King Abdulaziz University (KAU) was mild (M=0.91, SD=±1.04), whereas the overall mean percentage score of undergraduate nursing students' attitudes toward care, education, and advocacy for autistic children was 74.38%, indicating a positive high level of attitude.

Conclusions

This study highlighted the level of nursing students' knowledge about childhood autism. Additionally, it disclosed their attitudes toward autistic children. The study filled a gap in the literature by revealing the perspectives of nursing students toward ASD.

## Introduction

Autism spectrum disorder (ASD) is a developmental disorder. It is recognized as impairing socialization and communication and causing frequent and restricted unusual behaviors [1]. The prevalence of children diagnosed with ASD is increasing [1]. Many factors can contribute to the rise in ASD diagnoses, such as modern diagnostic measurements and specialized diagnostic tools. However, it is not clear whether detection and reporting have improved or whether the number of reported cases has risen.

Statistics from the King Abdulaziz City for Science and Technology show that the incidence rate of ASD among children in the Kingdom of Saudi Arabia (KSA) is one in every 180 children [2]. Males are four times more likely to be diagnosed with autism compared to females [2]. The reasons for developing autism remain unknown. Genetic and environmental factors may increase the risk of ASD [3]. One study found that mercury can affect metabolism, alter neuronal plasticity, and cause autism [2].

One study showed that supplementation with vitamin D and tryptophan among pregnant women is an affordable method for preventing ASD among children [2]. Similarly, another study showed that taking iron during pregnancy reduces the risk of developing autism [2]. Other risk factors for having autistic children are a relative's marriage and advanced maternal age [2].

Although various studies have been conducted on ASD worldwide, few have been conducted in the Middle East, especially in the KSA [3].

The ASD community faces numerous challenges, including economic challenges. They are also dealing with the rising cost of healthcare services at private institutions, increasing family burden, and social support from institutions [4]. In developed countries, there is a need for institutionalized financial support [1].

Nursing assessment and care must promote better patient outcomes, reduce family burdens, increase families' knowledge about how to deal with autistic children, and strengthen the family member and child relationship. This would lead to increased use of healthcare services and improve child gratification [2].

Another challenge that autistic children face is discrimination. Discrimination is due to a lack of knowledge about developmental needs and mental illness [2]. To resolve this issue, more emphasis should be placed on ASD policies by regulations and educational campaigns.

Poor knowledge of ASD among undergraduate nursing students might hinder children's progress and cause delays or uncertainties in providing appropriate nursing interventions. Thus, it is important for healthcare providers, including nursing students, to have adequate knowledge about ASD to provide an early diagnosis, carry out early intervention, and improve the life and well-being of autistic children.

Nursing students should have adequate knowledge about ASD to provide holistic care to autistic children. Nurses play a major role in the healthcare delivery system. They act as educators, care providers, and advocates, and they provide support to children and their families. However, if nursing students do not have enough knowledge about autism, the care they deliver will be inadequate. Thus, this study is essential to assess undergraduate nursing students' knowledge about and attitudes toward children with ASD, which is also its aim.

A cross-sectional descriptive study was conducted in Al-Taif City in KSA to assess undergraduate students' knowledge about and attitudes toward children with ASD [5]. A total of 247 medical students participated. Approximately 90% of sixth-year students knew more about autism compared to second-year students. The study recommended improving medical students' training practice and providing them with adequate clinical practice, including contact with ASD patients.

A study conducted in Turkey explored ASD awareness and knowledge of first-grade nursing and medical students [4]. A total of 175 students participated. A total of 138 participants had good knowledge of autism. A total of 81 students confirmed that "autism is a social and emotional developmental disorder"; out of these, 53% were nursing students, and 38.6% were medical students. The authors recommended further studies on this topic.

In Pakistan, a cross-sectional descriptive study was conducted to assess the level of knowledge about ASD among medical students [6]. A total of 157 students participated. The results showed that students in public universities had better knowledge compared to students in private universities. Medical students showed a lack of knowledge about ASD. To overcome this issue, the researcher recommended conducting further education.

A cross-sectional descriptive study was conducted in Turkey to assess the level of knowledge about children with autism among pharmacies [7]. A total of 141 pharmacies participated. Participants showed a lack of knowledge about the social features of autism. The authors recommended enhancing their knowledge and awareness of ASD, including its basic social features.

A cross-sectional descriptive study was conducted in Nigeria to assess medical doctors' knowledge of children with ASD [1]. The total number of participants was 175 (medical doctors, pediatricians, psychiatrists, and general practitioners). A total of 167 out of 175 participants had good knowledge of ASD. However, general practitioners showed poor knowledge of ASD. The authors recommended providing extra courses about ASD to educate non-specialists to perform therapeutic interventions in community settings [1].

A cross-sectional descriptive study was conducted in Pakistan to assess the degree of knowledge of ASD among healthcare professionals [8]. The total number of participants was 247 (physicians and nonphysicians). The results showed that the physicians and nonphysicians misunderstood the salient characteristics of ASD. Additionally, the results showed that low-income countries lack policies for diagnostic practices and have low knowledge about child mental health. The authors recommended improving participants' knowledge by providing important information about ASD [8].

A cross-sectional descriptive study was conducted in Nigeria to evaluate elements affecting knowledge about children with ASD among final-year undergraduate students [9]. The total number of participants was 300. The results showed that the level of knowledge among medical students was the highest, whereas that among psychology students was the lowest. The authors recommended that undergraduate psychology students be provided more lectures/courses to improve their knowledge about children with ASD [9].

Another cross-sectional descriptive study was conducted in Nigeria to recognize ASD manifestations and knowledge about some other types of ASD among final-year medical students [10]. A total of 757 final-year medical students participated. The results showed that, in general, there was a lack of knowledge of ASD among final-year students. However, participants who had previous clinical experience with children diagnosed with autism disorders had better knowledge. The authors recommended emphasizing ASD and neurodevelopmental disorders when teaching medicine. They also recommended emphasizing both during the psychiatry and pediatric clinical experience [10].

A quantitative study was conducted in Nigeria [9]. A total of 80 psychiatric and pediatric nurses participated. The results showed that psychiatric nurses have higher knowledge than pediatric nurses about children with ASD and that the former are more likely to recognize manifestations of children with autism compared to the latter. The authors recommended providing continuing medical education training to ensure early identification and diagnosis of ASD and conducting early intervention to improve prognoses [9].

A cross-sectional descriptive study was conducted in Brazil to assess nursing students' knowledge about autism disorder. The total number of participants was 65 [11]. The results showed a low level of knowledge about ASD and about symptoms and treatment of ASD. This knowledge is vital to provide ethical and evidence-based care. The study recommended bridging the gap by using technology. Media was identified as the main source of knowledge about ASD for participants [11].

In summary, previous studies revealed limited knowledge about ASD at all levels of the medical profession. However, most of these studies were conducted in countries other than the Middle Eastern or Arab countries. Thus, it is crucial to conduct further studies in the Middle East to assess nurses' knowledge about and attitudes toward autistic children. Nurses play an important role in providing care to children and their families. Therefore, they need to have enough knowledge about diagnostic criteria, assessments, and support measures.

## Materials and methods

Design and settings

A quantitative, analytical cross-sectional study was conducted at the Faculty of Nursing at King Abdulaziz University (KAU) in Jeddah, KSA.

Sample and sample size

The sample was a convenience sample. Data was collected in February 2019. Inclusion criteria includes students in the Faculty of Nursing at KAU as they were all eligible to participate. The population size was 392. Students not enrolled in the nursing programs were excluded from this study.

The sample size was calculated using Raosoft, Inc. (Seattle, Washington, United States). The inputs were as follows: margin of error, 5%; confidence level, 95%; and total student population, 392. The recommended minimal sample size was 195. The total number of students who agreed to participate was 235.

Ethical considerations

Ethical approval was obtained from the Research Ethical Committee of the Faculty of Nursing at KAU (approval number: 1F.24). The students of the faculty were not harmed, and their rights were respected and protected. Participants were told that their involvement in the study would be voluntary and that they had the right to not participate or to quit at any time. Researchers fulfilled their ethical duty of protecting participants' information and confidentiality. All questionnaires were anonymous. No personal identification was required from participants. Only the research team and the supervisor had access to the data.

Tool

The study used a questionnaire adapted from Helmy [5]. The tool was designed to assess students' level of knowledge about children with ASD and their attitudes toward ASD. The tool was an anonymous, self-administered questionnaire in the English language and consisted of three parts: sociodemographic; general knowledge about ASD (15 items); and attitudes of participants toward care, teaching, and support for children with ASD (five items). The tool was reviewed by experts in the field for face validity. Knowledge items were answered using "Yes or No" statements, whereas attitude items were answered using "Yes or No" statements. A higher overall score indicates a higher level.

Data collection procedures

Data was collected by the researchers through explaining the study, recruiting, and inviting students to participate in the study. The researchers distributed the paper-based questionnaires to all interested participants to be completed and placed in enclosed envelopes that were available in each class.

Data analysis

Analysis of data was conducted using IBM SPSS Statistics for Windows, Version 28.0 (Released 2021; IBM Corp., Armonk, New York, United States). Descriptive statistics such as frequencies and percentages were used to describe demographic data and to assess undergraduate nursing students' level of knowledge about children with ASD at KAU. T-independent samples test and one-way analysis of variance (ANOVA) test were conducted to measure the relationship between undergraduate nursing students' level of knowledge about children with ASD and their demographic characteristics.

## Results

A total of 235 students participated in the study, achieving a response rate of 59.5%. Table 1 demonstrates the participants' demographic characteristics.

**Table 1:** Participants' demographic characteristics

Item	N	%
Age		
Less than 20 years	41	17.45%
20 years and over	194	82.55%
Faculty grade		
Second year	120	51.1%
Third year	40	17%
Fourth year	75	31.9%

As Table 1 shows, the age of the majority of the studied sample was 20 years and over (n=194, 82.55%). The rest of the sample was less than 20 years old (n=41, 17.45%). The majority of the students were in their second year (n=120, 51.1%). The lowest number of students were in their third year (n=40, 17%).

Table 2 shows the distribution of the percentage and frequency of the knowledge items among undergraduate nursing students. The total mean score of the overall knowledge levels among nursing students at KAU was mild (M=0.91, SD=±1.04). The mean was less than 1, indicating that the knowledge and attitude levels among nursing students in KAU were mild.

**Table 2:** Knowledge about children with ASD ASD: autism spectrum disorder

Have you heard about ASD?	Frequency	Percent
Yes	201	85.5%
No	34	14.5%
Total	235	100%
Have you seen a child with autism?		
Yes	156	66.4%
No	79	33.6%
Total	235	100%
Have you attended any campaigns about autism?		
Yes	19	8.1%
No	216	91.9%
Total	235	100%
Can poor parenting practices cause ASD?		
Yes	142	60.4%
No	89	37.9%
Total	231	98.3%
Does ASD likely result from complex environmental, neurological, immunological, and genetic factors?		
Yes	209	88.9%
No	22	9.4%
Total	231	98.3%
Do genetic factors play an important role in or cause autism disorders?		
Yes	167	71.1%
No	62	26.4%
Total	229	97.5%
Can autism disorder be diagnosed by medical methods?		
Yes	130	55.3%
No	101	43%
Total	231	98.3%
Are children with autism frequently diagnosed depending on their physical features?		
Yes	134	57%
No	97	41.3%
Total	231	98.3%
Autistic children have problems in the following areas:		
Social interaction	9	3.8%
Social interaction and speech	6	2.6%
Social interaction, speech, and behaviors	210	89.4%
	225	95.8%
A total of 10% of autistic children have extraordinary skills		
Yes	196	83.4%
No	34	14.5%
Total	235	100%
Are the majority of autistic children girls?		
Yes	16	6.8%
No	214	91%
Total	230	97.8%
Can the majority of autistic children not play an imaginary type of play?		
Yes	92	39.1%
No	142	60.4%
Total	234	99.5%
Do autistic children not prefer routine activities?		
Yes	114	48.5%
No	120	51.1%
Total	234	99.5%
Are behavioral interventions considered the most effective treatment method for autism?		
Yes	217	92.3%
No	10	4.3%
Total	227	96.6%
With proper intervention, can children with autism disorder eventually outgrow the disorder?		
Yes	158	67.2%
No	68	28.9%
Total	226	96.1%

Additionally, two-thirds of the participants (n=142, 60.4%) believed that poor parenting practices can cause ASD. The majority of participants (n=209, 88.9%) believed that ASD likely results from complex environmental, neurological, immunological, and genetic factors. Nearly three-quarters of the participants (n=167, 71.1%) believed that genetic factors play a significant role or are a risk factor in autism disorder.

A total of 130 (55.3%) participants knew that autism disorder is diagnosed using medical methods, whereas other participants believed that children with autism can be diagnosed depending on their physical features (n=134, 57%). A total of 210 (89.4%) of autistic children had problems with social interaction, speech, and behavior; 3.8% had problems with social interaction; and 2.6% had problems with social interaction and speech. The majority (n=196, 83.4%) of the sample agreed that 10% of autistic children can have extraordinary skills.

Moreover, 142 (60.4%) of the participants did not agree that the majority of autistic children cannot play an imaginary type of play, and 120 (51.1%) did not agree that autistic children do not prefer routine activities. Finally, the majority of the participants (n=158, 67.2%) believed that with good intervention, most autistic children will eventually outgrow the disorder.

Table 3 shows the undergraduate nursing students' attitudes toward care, education, and advocacy for autistic children, of which the overall mean percentage score was 74.38%. This indicated a positive high level of attitude.

**Table 3:** Undergraduate nursing students' attitudes toward care, education, and advocacy for autistic children

Factors	Yes		No		No answer	
Should autistic children not be integrated into mainstream schools?	91	38.7%	136	57.9%	8	3.4%
Do you think there is discrimination against autistic children in Saudi Arabian society?	170	72.3%	56	23.8%	9	3.8%
Will you permit your son or daughter to play with a child having autism?	191	81.3%	35	14.9%	9	3.8%
Should the government allocate more resources for the provision of services to children with autism?	211	89.8%	14	6%	10	4.3%
Should more campaigns and workshops be conducted to increase the community's knowledge of autism?	211	89.8%	16	6.8%	8	3.45%
Overall percentage (total mean score)	-	74.38%	-	21.88%	-	-

Relationship between undergraduate nursing students' level of knowledge about ASD and their demographic characteristics

To measure the relationship between undergraduate nursing students' level of knowledge about ASD and their demographic characteristics, the researchers conducted a T-independent samples test with age and a one-way ANOVA test with faculty grade. Table 4 shows the results.

**Table 4:** Statistics of the relationship between undergraduate nursing student levels' of knowledge about ASD and their demographic characteristics ASD: autism spectrum disorder

Age	N	Mean score	± Std.	T-value	P-value
Less than 20 years	41	24.73	4.48	0.210	0.834
20 years and over	194	24.83	2.39		
Faculty grade	N	Mean score	± Std.	F-value	P-value
Second year	120	25.15	3.19	1.815	0.165
Third year	40	24.28	2.31		
Fourth year	75	24.57	2.52		

The statistics of the relationship between undergraduate nursing students' level of knowledge about children with ASD and their demographic characteristics are presented in Table 4. No significant relationship was found (T-value=0.210; p-value=0.834). Similarly, no significant relationship was found between undergraduate nursing students' level of knowledge about childhood ASD and faculty grade (F-value=1.815; p-value=0.165).

The statistics for the relationship between undergraduate nursing students' attitudes toward care, education, and advocacy for autistic children and their demographic characteristics are shown in Table 5. It was found that age did not show any significant relationship with the undergraduate nursing students' attitude related to care, education, and advocacy for autistic children, where the T-value is 1.515 and the p-value is 0.131.

**Table 5:** Statistics of the relationship between undergraduate nursing students' attitudes toward care, education, and advocacy for autistic children and their demographic characteristics

Age	N	Mean score	± Std.	T-value	P-value
Less than 20 years	41	6.80	1.89	1.515	0.131
20 years and over	194	7.23	1.58		
Faculty grade	N	Mean score	± Std.	F-value	P-value
Second year	120	7.1917	1.47981	0.779	0.460
Third year	40	7.3750	1.16987		
Fourth year	75	6.9867	2.06315		

Also, similarly, the faculty grade variable did not show any significant relationship with the undergraduate nursing students' attitudes related to care, education, and advocacy for autistic children, where the F-value is 0.779 and the p-value is 0.460.

## Discussion

The results showed no significant relationship between participants' education level and knowledge about or attitudes toward ASD. Previous studies showed that students obtain better knowledge as they gain more clinical experience. Additionally, specialists who have more interaction with autistic children have better knowledge of ASD. A Turkish study found that nursing students had better knowledge of ASD compared to medical students [4] and that pharmacists had the least amount of knowledge [7]. This could be related to the nursing student's knowledge levels regarding children with ASD; unlike other specialists, pharmacists do not take pediatric courses and do not have medical experience with autistic children. It might be necessary to conduct another study with a larger sample of students from different health specialties to assess their knowledge and attitudes.

In our study, we found that 85.5% of participants had heard about ASD. However, in another study conducted among nursing students in Brazil, 65 undergraduate nursing students had limited knowledge about ASD [11]. Two studies were conducted in Nigeria. One was conducted among final-year medical students, and the other was conducted among medical students [10]. The studies showed that students lacked knowledge about ASD. Only those with previous exposure to autistic children had better knowledge of ASD [10]. The second study was conducted among final-year medical, nursing, and psychology students [9]. Consistent with the current study, the results showed that medical students had the best knowledge about ASD, followed by nursing students, whereas psychology students had the lowest amount of knowledge [9]. However, the previous study revealed that psychiatric nurses had better knowledge of ASD than pediatric nurses [9]. In another study conducted in Nigeria among medical doctors, pediatricians, psychiatrists, and general doctors, the results showed that most of them had good knowledge about the causes of ASD. General practitioners had the lowest amount of knowledge of ASD [1]. In a study conducted in Pakistan among medical students from private and public universities, the results showed that students from public universities had better knowledge than students from private universities [6]. In another study conducted in Pakistan among physicians and nonphysicians, there was some misunderstanding about the salient characteristics of autism among participants [8]. The authors recommended providing extra courses on the clinical features of ASD.

In our study, 66.4% of participants had seen a child with autism. However, in a study conducted in Al-Taif, 54% of participants had not seen a child with autism [5]. It is essential that participants see and interact with autistic children to recognize their characteristics and learn how to deal with them.

In our study, 91.9% of participants did not attend any campaign about ASD, and in the Al-Taif study, sixth-year students did not attend any campaigns about ASD [5]. It is essential that participants attend such campaigns to increase their awareness of and interaction with the autistic community.

In the current study, 71.1% of participants knew that autism is a genetic disorder. In the Al-Taif study, all of the neuropsychiatrist residents knew that autism is a genetic disorder [3]. In the latter study, sixth-year medical students (61.3%) knew that autism is a genetic disorder, whereas only second-year medical students (16.7%) knew autism is a genetic disorder [5]. These results show the importance of including more topics and discussions relating to autism in the nursing curriculum.

From the literature, it was evident that few studies have been conducted in KSA among nursing students. Thus, the current study filled an important gap. Nurses are the first point of contact with patients, and they play a major role in supporting patients and their relatives. It is hoped that this research will help ensure that nursing students have comprehensive knowledge about autism in the future. Professional nurses can use this study's results to develop an effective educational program and to provide appropriate diagnosis and treatment.

Limitations

This study has some limitations. First, only female students participated in the study. Second, the data collection method involved a self-administered questionnaire, which is subject to bias. Also, there is a lack of generalizability as the data is collected from a single setting.

## Conclusions

Nursing students have little knowledge about ASD, and this knowledge is obtained mostly from the media. Additionally, the results of this study showed that most participants had heard about ASD and had seen a child with autism in their community. Most participants believed that ASD likely results from poor parenting and genetic factors. Some participants believed that most autistic children are girls. Regarding attending campaigns about ASD, almost none of the participants had attended such a campaign. Integrating comprehensive ASD awareness training into nursing education will equip future healthcare professionals with the knowledge, skills, and empathy needed to provide competent and compassionate care to children with ASD and their families.
